# Strengths-Based Nursing to Combat Common Infectious Diseases in Indigenous Australians

**DOI:** 10.3390/nursrep12010003

**Published:** 2022-01-18

**Authors:** Rajkumar Cheluvappa, Selwyn Selvendran

**Affiliations:** 1 Nursing and Midwifery, Australian Catholic University, Watson, ACT 2602, Australia; 2 Department of Surgery, St. George Hospital, Kogarah, NSW 2217, Australia; tselvendran@hotmail.com

**Keywords:** Aboriginal, chlamydia, gonorrhoea, HIV, Indigenous Australians, infectious disease, Strengths-Based Nursing, scabies, skin infection, skin sore, syphilis

## Abstract

(1) Problem: The increasing incidence and prevalence of infectious diseases in Indigenous Australians (Aboriginal groups and Torres Strait Islanders) are concerning. Indigenous Australians experience the burden of infectious diseases disproportionately when compared to non-Indigenous Australians. (2) Aim: Our report aims to describe how to apply Strengths-Based Nursing (SBN) to ameliorate the impact of the most common infectious diseases in Indigenous Australians. Specifically, we aim to describe how nurses can use SBN to partner with Indigenous Australian communities to remediate, control, and mollify the impact of the most common infectious diseases encountered by them using their limited resources. (3) Methods: Meticulous PubMed, Google Scholar, and web searches were conducted pertaining to Strengths-Based Nursing and common infectious diseases in Indigenous Australians. (4) Findings: The two groups of infectious diseases considered are sexually transmitted infections (STIs) and infectious skin diseases (including parasitic infestations). The prevalence of these infectious diseases in Indigenous Australians is deliberated on, with data when possible, or known trends and impacts. Finally, existing, evidence-based, prudent, and possible SBN approaches are discussed towards tackling these infectious diseases judiciously with available local resources, in conjunction with the support of impacted people, their families, and their communities. (5) Discussion and Conclusion: The SBN approach is a relatively new perspective/approach to clinical and nursing care. In contradistinction to the commonly utilised medical model, SBN pits strengths against deficits, available resources against professional judgment, solutions against unavailable items, and collaborations against hierarchy. In light of the current situation/data, several SBN approaches to combat STIs and skin infections in Indigenous Australians were identified and discussed for the first time in the “Results” section of this paper.

## 1. Introduction

### 1.1. Clinical Relevance of This Paper

#### 1.1.1. Problem or Issue

The incidence and prevalence of infectious diseases in Indigenous Australians are increasing.

#### 1.1.2. What Is Already Known

Indigenous Australians experience the burden of infectious diseases disproportionately when compared to non-Indigenous Australians.

#### 1.1.3. What This Paper Adds or Elaborates On

Using Strengths-Based Nursing (SBN), nurses can partner with Indigenous Australian communities to remediate, control, and mollify the impact of STIs and infectious skin diseases on them using their limited resources. Several SBN approaches are discussed towards tackling these infectious diseases judiciously with available local resources, in conjunction with the support of impacted people, their families, and their communities.

### 1.2. Strengths-Based Nursing

**Figure 1:**
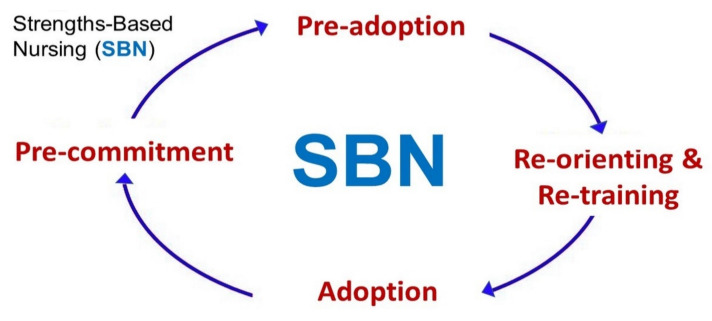
Steps involved in Strengths-Based Nursing (SBN).

The Strengths-Based Nursing (SBN) approach analyses target group or organisational needs, utilises the strengths of group/organisation-associated individuals to promote tailored self-sufficiency, efficiency, and empowerment of patients or clients/participants [1]. The SBN approach guides academics, medical administrators, clinicians, and nursing personnel in their professional practices and in appropriate strategising to achieve their targets efficiently and benevolently [2]. When possible, it involves available family members of individuals in need of care [3]. For practical purposes, the SBN approach may be divided into two phases, the pre-commitment/pre-adoption phase, and the adoption phase (Figure 1) [4]. Even when facing challenging healthcare circumstances, SBN providers can identify gaps, locate bridging resources, harness help from concerned community members, and promote skills that patients and their family need to remedy healthcare issues for different periods of time. Therefore, SBN providers may need to have managerial and human resources skills in addition to clinical expertise [1].

To ensure the success of SBN, meticulous planning, consultation, flexibility, and resourcefulness are essential. It is key to ensure that relevant clinical personnel should have an impelling desire to utilise SBN, find a minimum number of like-minded co-workers, and make the SBN approach patient-focussed, resource availability-centred, and fine-tuned to the community context, environment, and circumstances (Figure 1) [4].

## 2. Materials and Methods

The most common infectious diseases (incidence and prevalence) in Indigenous Australians were identified by an initial examination of the authoritative Australian Institute of Health and Welfare (AIHW) website and Commonwealth Scientific and Industrial Research Organisation (CSIRO) reports. Verification was conducted by using more recent news reports and information from the Kirby Institute, University of New South Wales (UNSW). From these cited sources, we narrowed down the most common infectious diseases in Indigenous Australians to include only two groups, STIs and infectious skin diseases, and exclude the rest. Word searches pertaining to these two groups included combinations of pertinent words like syphilis, gonorrhoea, chlamydia, HIV, AIDS, scabies, sores, skin, lesions, infections, infectious, public health, epidemiology, Aboriginal, Torres Strait Islanders, Australia, Australian, and Indigenous. Searches were conducted using PubMed, Google Scholar, and government reports. References from publications resulting from these sources were also investigated for relevant data or information suitable for the SBN approach. Targeted searches were conducted on PubMed for programs, protocols, and approaches to tackle STIs and infectious skin diseases in Indigenous Australians. Topically relevant general review articles were excluded. Original research publications using programs, protocols, and approaches showing statistical significance and logistical promise were further dissected. This process of honing resulted in around 20 publications which informed our report.

## 3. Results

### 3.1. Search Outcomes

The selection, characteristics, and synthesis of studies, reports, data, and information were described in the previous section. There were around 70 publications related to the topics spread out over the last 20 years, but most had sparse data or just anecdotal information. Out of the ~70 publications, only ~20 contained data relevant to the topics discussed herein. These 20 publications included epidemiology papers, meta-analyses, and papers detailing pilot projects (protocols and programs). Australian government sources and media reports were also sourced for the latest data and trends pertaining to skin infections and STIs in Indigenous Australians.

### 3.2. Infectious Diseases in Indigenous Australians

The increasing incidence and prevalence of infectious diseases in Australian Aboriginal and Torres Strait Islander populations is disconcerting [5,6]. Indigenous populations experience the burden of infectious diseases disproportionately when compared to non-Indigenous Australians [7,8].

Sexually Transmissible Infections/diseases (STIs) are a major concern in younger Indigenous Australians [8]. The rates of Human Immunodeficiency Virus (HIV) notification have doubled during the second half of the previous decade and are increasing [7]. A recent meta-analysis including data from seven individual cross-sectional studies corroborates this finding [9]. Notification rates of chlamydia in Indigenous Australians are thrice those of non-Indigenous Australians [10,11]. Notification rates of gonorrhoea in Indigenous Australians are ten times greater than those of non-Indigenous Australians [10,12]. Notification rates of infectious syphilis (primary and secondary syphilis) in Indigenous Australians are six times greater than those of non-Indigenous Australians [11]. Recently, a decade-long syphilis outbreak amongst young Indigenous Australians from the Northern Territory (NT) has crossed borders and is spreading through parts of Queensland, Western Australia, and South Australia [8,13].

In the East Arnhem region of the NT, there is an unacceptably high prevalence of transmissible skin infections (20%) and skin sores (15%) as shown by combined data from three separate publications [6]. From the same datasets, it was evident that 95% of children had received at least one antibiotic and almost 50% of children had received six antibiotic prescriptions by the first year of life [6]. This is extraordinarily high. This is not only concerning in the interests of Indigenous Australian health, but also in the dire necessity to curb antibiotic resistance by curtailing antibiotic misuse or overuse (antibiotic stewardship) [14,15]. In a recent retrospective study of children under 5 years from the Western Desert region of Western Australia (WA), data showed that 72% of children presented at least once with skin infections and 2% of children presented with scabies, an arthropod parasitic infection. Out of the 72% of children presenting with skin infections, 75% presented with skin sores [5].

### 3.3. Pre-Commitment to Implement

The successful evaluation of SHIMMER, a regional New South Wales (NSW) sexual health quality improvement program (QIP), was gratifying in that it tripled the testing of gonorrhoea and chlamydia in young Indigenous Australians and doubled the detection of chlamydia infections [10]. The existing, established South Australian (SA) STI testing program commenced in 2008/2009 and was implemented at nine Aboriginal Community Controlled Health Services. The South Australian data collected from 2008 to 2016 demonstrated that STI testing increased with time and positivity for STIs decreased, but only in females [16]. This implies that STI testing should be significantly increased in males to improve detection. The Test Treat ANd GO (TTANGO) protocol using a molecular Point Of Care (POC) testing methodology is suitable for testing remotely located Indigenous Australians for gonorrhoea and chlamydia and immediately giving the results [17]. These can be used with great efficiency by recruiting and training local social workers, family members, and community representatives as part of efficient SBN (Figure 1). Community-centred, culturally tailored programs which are focused on prevention for individuals at risk and supportive management of people who have HIV infection are needed to curb the increasing incidence and prevalence of HIV in their respective population groups [9]. The Kirby Institute has strong partnerships with Aboriginal and Torres Strait Islander organisations and services which can be utilised towards SBN via the availability of a readymade list of hand-picked community representatives and volunteers [7].

The See Treat Prevent (SToP) protocol uses three intervention steps to manage skin infections, sores, and scabies infestations [18]. The three steps are seeing and identifying skin infections and/or scabies (after community dermatology model training), treating using evidence-based approaches, and preventing via local health education promotion to hand-picked community members (SBN) and adjustments to the local environment (Figure 1) [18]. Clinical examination is the main diagnostic approach for scabies whilst dermoscopy scraping microscopy can also be performed [19]. Secondary bacterial infections and skin sores are common sequalae to scabies skin infestation. Topical permethrin is commonly used to treat individual cases, and treatment with oral ivermectin is used for controlling outbreaks or managing asymptomatic contacts and control of outbreaks [19]. Identifying, recruiting, and training local community members and leaders to assist in identifying and reporting suspicious skin lesions would be a good SBN approach.

### 3.4. Implementation via Adoption

Nurses should first identify and promote representatives from the community to participate in the SBN initiative. Such representatives or volunteers should have specific capabilities, knowledge, and flexibility in skill-acquiring suited to the purpose at hand [1]. The resources and options/decisions of a patient and his/her family and well-wishers should be identified as SBN is expected to be patient-centred and family-centred [1]. The curating of trimmed-down information and instructions to transmit to them is equally essential. The fine-tuning of time and location logistics, and the tactful use of scarce resources is crucial to promote health using SBN [1].

A key preventative SBN approach is to test young Indigenous Australians for STIs to detect asymptomatic infections early and manage it with antibiotics and education. Crucially, contact tracing and mandatory notification (when relevant) to public health authorities are essential for prevent or mitigate outbreaks. Using SBN, nurses in conjunction with local volunteers could use the SHIMMER protocol to scan for and test these two STIs in primary health centres and improvised meeting places allocated by the local Indigenous community leaders [10]. Nurses can utilise SBN to train resourceful community members and extended family members of affected individuals to go to “inaccessible” places and use the TTANGO protocol using POC testing for gonorrhoea and chlamydia. Nurses, social workers, and community members can seek the help of local Indigenous leaders for contact tracing assistance and to reach out and screen people for these STIs via home visits. Local volunteers recruited via SBN can also provide culturally appropriate community-based clinical management, medication compliance, and regular follow-up for Indigenous Australians, especially for those living in remote locations (Figure 1). This is essential not only to control gonorrhoea and chlamydia, but also to impede the growing incidence and prevalence of HIV infections in Indigenous Australians [9].

Using the SToP protocol to eliminate or decrease skin infections can reduce the misuse of antibiotics from different antibiotic groups, decrease microbial antibiotic resistance, and conserve/preserve antibiotics for when they are really needed in the interests of antibiotic stewardship [15]. This can be done in conjunction with SBN-identified community of family recruits in flexible community meeting places allocated by local leaders. The SBN strategy of using local volunteers is crucial to ensuring compliance to skin treatment protocols and regular follow-up Indigenous Australians living in remote groups (Figure 1) [20]. Distribution of ivermectin (for scabies outbreaks) to remote places may be performed by SBN-enlisted trained community recruits and family members.

## 4. Discussion

### 4.1. Key Findings in This Report

If implemented more widely, the SHIMMER program could double/triple the testing of gonorrhoea and chlamydia in young Indigenous Australians. The TTANGO protocol will aid giving quicker results for gonorrhoea and chlamydia in remote Indigenous Australians. Detection of STIs may improve if STI testing is increased in Indigenous males. The existing partnerships that the Kirby Institute has with Indigenous organisations/services and their prepared community representatives/volunteers will facilitate using SBN to detect and manage HIV cases. Using SBN, local volunteers can give culturally appropriate community-based treatment, ensure compliance, and arrange follow-up for Indigenous Australians to control gonorrhoea, chlamydia, and HIV. The SToP protocol can be used to detect and manage skin infections, sores, and scabies infestations. Unfortunately, owing to ambit limitations, SBN approaches may not be able to tackle the current syphilis outbreak amongst young Indigenous Australians from NT and its bordering areas.

### 4.2. Limitations to Using Strengths-Based Nursing to Combat Infectious Diseases in Indigenous Australians

The main limitation to nurses using SBN to partner with the Indigenous Australian community to battle the common infectious diseases in Indigenous communities is adequate funding from the government, private donors, philanthropic organisations, Indigenous bodies, native title body leaders, and Non-Governmental Organisations (Figure 1). Whilst it may be argued that one of the purposes of SBN is to cut down the need for moderate or substantial funding, SBN still needs a minimum amount of funding to operate.

An operational limitation is the literacy level of individual local community volunteers and family members because the logistics of several SBN operations are dependent on their levels of comprehension and their capacity to be trained to carry our specific tasks. Local volunteers need to be appreciated with incentives and recognition and, to some extent, compensated for their time and efforts in cash or kind.

Regretfully, SBN approaches cannot tackle, even minimally, the ongoing decade-long syphilis outbreak amongst young Indigenous Australians from NT and its bordering areas because of several logistical, geographical, financial, and jurisdictional difficulties associated with syphilis pathophysiology, clinical presentations, diagnostic testing, and treatment [8,13]. A thorough discussion of these is out of scope of this submission topic.

Owing to the exploratory nature of this SBN-undergirded report and the spectrum of possible approaches available to manage infectious diseases in Indigenous Australians, it is premature to assess bias in the studies included herein. However, it is possible that the remoteness of several Indigenous Australian communities may underestimate notification rates of STIs, underscoring the utility of the SBN approach.

### 4.3. Challenges to Using Strengths-Based Nursing to Combat Infectious Diseases in Indigenous Australians

Several challenges remain. Recent increases in the incidence and prevalence of gonorrhoea, syphilis, HIV, and chlamydia infections in Indigenous Australians, when compared to non-Indigenous Australians, are a major challenge to surmount [21]. Antibiotic resistance against several groups of antibiotics is increasing in Australia [21,22]. A recent study using a previously validated evaluation tool retrospectively assessed randomly selected antibiotic prescriptions given between 1 January 2018 and 31 December 2018 for mostly skin and soft-tissue infections (71%) in ten central Australian Aboriginal clinics [23]. This study showed that 25.7% of prescriptions were inappropriate based on the validated evaluation tool criteria [23]. The same study also showed that 44.4% of prescriptions, especially those written by general practitioners practising in remote communities, did not conform to established guidelines [23]. Systemic and systematic government and Australian Health Practitioner Regulation Agency (AHPRA) policy changes need to be made and new mandatory protocols need to be instituted for this as SBN alone cannot resolve these issues without political will, legislative backing, and funding [22].

## 5. Conclusions

The SBN approach discussed in this paper uses locally available strengths and resources by partnering with local volunteers and recruits from the local Indigenous Australian communities. The infectious disease groups dealt with herein were STIs and skin infections. The specifics and burdens imposed by these infectious diseases on Indigenous Australians were discussed with evidence, taking care not to digress into infectious diseases outside these two groups. Prevention and management of these infectious diseases in Indigenous Australians using SBN were elaborately discussed. In particular, a few ways to use SBN to harness existing, evidence-based detection and management approaches were discussed with the objective of using minimum local resources towards tackling these infectious diseases with the support of locally recruited family members and community representatives. The limitations of using SBN and the possible challenges that may be encountered whilst using SBN to tackle infectious diseases in Indigenous Australians were finally discussed.

## Data Availability

Relevant raw data is available from the references cited.

## Abbreviations

AIHW: Australian Institute of Health and Welfare; HIV, Human Immunodeficiency Virus; NSW, New South Wales; NT, Northern Territory; POC testing, Point Of Contact testing; QIP, Quality Improvement Program; QLD, Queensland; SBN, Strengths-Based Nursing; STI, Sexually Transmitted Infection; SToP protocol, See Treat Prevent protocol; TTANGO protocol, Test Treat ANd GO protocol; UNSW, University of New South Wales; WA, Western Australia.
